# Preferred Parental Language and Neurodevelopmental Outcomes Among Infants With Acute Provoked Neonatal Seizures in the United States

**DOI:** 10.1016/j.pediatrneurol.2024.12.010

**Published:** 2024-12-26

**Authors:** Greta S. Peng, Karin Halsey, Courtney J. Wusthoff, Catherine J. Chu, Shavonne L. Massey, Monica E. Lemmon, Cameron Thomas, Adam L. Numis, Giulia M. Benedetti, Julie Sturza, Elizabeth E. Rogers, Linda S. Franck, Charles E. McCulloch, Janet S. Soul, Renée A. Shellhaas, Sonia L. Bonifacio, Hannah C. Glass

**Affiliations:** a Department of Pediatrics, UCSF Benioff Children’s Hospital, University of California, San Francisco, San Francisco, CA; b Johns Hopkins Bloomberg School of Public Health, Baltimore, MD; c Department of Neurology, UC Davis Children’s Hospital, University of California Davis, Sacramento, California; d Department of Neurology, Massachusetts General Hospital, Harvard Medical School, Boston, Massachusetts; e Department of Pediatrics, Children’s Hospital of Philadelphia, Perelman School of Medicine at the University of Pennsylvania, Philadelphia, PA; f Department of Neurology, Children’s Hospital of Philadelphia, Perelman School of Medicine at the University of Pennsylvania, Philadelphia, PA; g Departments of Pediatrics, Duke University School of Medicine, Durham, North Carolina; h Department of Population Health Sciences, Duke University School of Medicine, Durham, North Carolina; i Division of Neurology, Department of Pediatrics, Cincinnati Children’s Hospital Medical Center, University of Cincinnati, Cincinnati, Ohio; j Department of Neurology and Weill Institute for Neuroscience, University of California, San Francisco, San Francisco, CA; k Department of Pediatrics, University of Michigan, Ann Arbor, MI; l Department of Family Health Care Nursing, University of California, San Francisco, San Francisco, CA; m Department of Epidemiology & Biostatistics, University of California, San Francisco, San Francisco, CA; n Department of Neurology, Boston Children’s Hospital, Harvard Medical School, Boston, Massachusetts; o Department of Neurology, Washington University in St Louis, St Louis, Missouri; p Division of Neonatal and Developmental Medicine, Department of Pediatrics, Stanford University, Palo Alto, California

**Keywords:** Neonatal seizures, Neurodevelopment, Parental language preference, Non-English language preference, Limited English proficiency, Spanish language preference, Language-related health inequities, Health equity

## Abstract

**Background:**

Parental non-English language preference (NELP) is associated with worse pediatric health outcomes. However, little is known about its relationship with developmental outcomes in infants with neonatal seizures. This study evaluated the relationship between parental NELP and neurodevelopment in a multicenter cohort of infants with neonatal seizures.

**Methods:**

Infants in the *Neonatal Seizure Registry*-II were included. Parental NELP was defined by the use of a professional interpreter for research consent and survey completion. The Warner Initial Developmental Evaluation of Adaptive and Functional Skills (WIDEA-FS) assessment was conducted at age 24 months. Multivariate regression was used to examine the association between parental NELP and WIDEA-FS. Functional developmental impairment was defined as a WIDEA-FS score 2 S.D.s below the normative mean.

**Results:**

Among 270 infants with neonatal seizures, 15 (6%) had parental NELP. Children with parental NELP had a WIDEA-FS score that was on average 13 points lower than that of infants without parental NELP (95% confidence interval [CI]: −27 to 1, *P* = 0.08) and over five times the odds of functional developmental impairment (odds ratio 4.9, 95% CI: 1.3 to 18.4, *P* = 0.017).

**Conclusions:**

Children with parental NELP were more likely to have functional developmental impairment at age 24 months when compared with children without parental NELP. Since parental NELP does not have a biologically plausible impact on neurodevelopment it likely reflects discriminatory experiences that affect developmental opportunities. These findings highlight the importance of identifying social drivers to decrease potential gaps in neurodevelopmental attainment for children with parental NELP.

## Introduction

The concept of limited English proficiency, also referred to as non-English language preference (NELP), was incorporated into US federal law in 2000 and formed the basis of numerous policy decisions across public and medical services.^1^ Based on US census data from 2019, nearly 8% of the population aged five years or older, approximately 25.1 million people, qualify as NELP (defined as individuals who do not speak English as their primary language and who have a limited ability to read, speak, write, or understand English).^2^ Additionally, 21.5% of US residents speak a language other than English at home and 13.2% of US residents primarily speak Spanish at home.^3^ Although many US health care institutions offer professional medical interpreting services, there are ongoing inequities in clinical outcomes and access to care due to a variety of factors including lack of access to professional interpretation, inadequate time during clinical encounters to account for interpretation, and suboptimal communication with providers.^4–6^

Parental NELP is associated with worse pediatric health outcomes, including longer hospitalizations, increased risk of serious adverse medical events, and decreased number of home health care referrals.^7–11^ Among children with special health care needs, parental NELP is associated with lower caregiver satisfaction, worse health care access, and poorer coordination of care.^12,13^ For children who require admission to the neonatal intensive care unit (NICU), some of the inequities in outcomes have been attributed to poorer communication during the hospitalization and increased inequities related to discharge and follow-up services such as lack of knowledge of developmental support resources.^14–19^ Parental NELP does not have a biologically plausible impact on brain development and therefore is likely a proxy for structural inequities and discriminatory experiences that impact health and developmental opportunities.

Infants treated in the NICU for acute provoked neonatal seizures may require access to longitudinal health care and follow-up that puts patients with parental NELP at risk for poorer clinical outcomes. Little is known about the association between parental NELP and neurodevelopmental outcomes in children. Existing data highlighting gaps in access to longitudinal health care follow-up and poor communication with health care professionals raise the possibility that infants with parental NELP are at risk of poorer neurodevelopmental outcomes. The goal of this study was to evaluate the relationship between parental NELP and performance on a functional neurodevelopmental screening assessment in a multicenter cohort of infants with acute provoked neonatal seizures. We hypothesized that infants with parental NELP would have lower scores compared with infants whose parents have an English language preference.

## Materials and Methods

A secondary analysis was performed on a prospective multicenter cohort study (NCT02789176) of neonates with acute provoked seizures born between July 2015 and March 2018 who were treated at one of the nine centers of the *Neonatal Seizure Registry*-II.^20^ Each center has a level IV NICU and a level IV comprehensive pediatric epilepsy program that follows the 2011 American Clinical Neurophysiology Society guidelines for continuous electroencephalography (EEG) monitoring.^21^ The clinical teams at each institution followed local protocols for the management of seizures. There was no specific treatment pathway or protocol recommended in this study. The local institutional review board at each site approved the study, and parents provided written informed consent in their preferred language.

### Inclusion criteria

Infants enrolled in the *Neonatal Seizure Registry*-II were included. One parent or legal guardian per family completed a suite of validated survey instruments near the time of discharge from the NICU and when their child reached 12, 18, and 24 months corrected age. Parents completed the surveys online or by telephone interview with a trained research assistant. Parents with NELP had access to professional medical interpreters during the consent process and during any interactions with a clinical research coordinator.

Enrollment criteria were as follows: (1) neonates with EEG-confirmed seizures or neonates who were treated with antiseizure medication for diagnosed or suspected seizures, (2) seizures that had an acute symptomatic etiology (i.e., hypoxic-ischemic encephalopathy, ischemic stroke, intracranial hemorrhage, or other brain injury), and (3) infants who survived the neonatal seizure admission. Neonates with transient cause for seizures (e.g., hyponatremia, hypocalcemia, hypoglycemia without brain injury) or neonatal-onset epilepsy syndromes were excluded.

### Primary variable

Parental NELP was determined by documented use of a medical interpreter for research consents or communications (i.e., interpreter signature on consent form, clinical research coordinator documentation of interpreter) and completion of survey instruments.

### Outcome variables

The primary outcome variable was the Warner Initial Developmental Evaluation of Adaptive and Functional Skills (WIDEA-FS) score at corrected age 24 months, which was conducted in the parents’ preferred language by a research coordinator who was not aware of the medical history other than the history of neonatal seizures. The WIDEA-FS is a 50-item questionnaire with a score ranging from 50 to 200 and is designed to assess adaptive skills in the domains of mobility, communication, social cognition, and self-care. A child was considered to have functional developmental impairment when their WIDEA-FS total score was more than 2 S.D.s below the normative mean for age.^20^

Covariates were infant sex assigned at birth, race/ethnicity, gestational age at birth, Apgar score at five minutes, presence of treatment with therapeutic hypothermia, days of EEG-confirmed seizures, initial EEG background pattern (normal/mild, moderate, or severe abnormalities, status epilepticus), effectiveness of initial antiseizure medication for seizure control (including loading dose effectiveness and whether the infant required two or more medications for seizure control), seizure etiology, neurological examination at discharge, NICU length of stay, and maternal education. Insurance type (public or private) was explored as a covariate, but due to collinearity with NELP status, it could not be included in the model. Covariate data were available for all participants in the primary analysis (i.e., WIDEA-FS as the primary outcome variable). In the analysis regarding special services, there was a higher percentage of missing data (16% of participants lacked data on the special services outcome). Access to developmental services at 24 months was assessed through a survey completed by a primary caregiver.

### Statistical analysis

To test for the statistical significance of the variables between the two groups, we used Fisher exact test for binary and categorical variables and Mann-Whitney *U* test for continuous variables. A *P* value of less than 0.05 was considered statistically significant. Multivariate regression (linear for WIDEA-FS score and logistic for the categorical outcome of functional developmental impairment) was used to examine the association between NELP and performance on the WIDEA-FS. Multivariate models were refined by removing nonsignificant covariates from the full model in a backward stepwise fashion until all remaining covariates were significant contributors to the model. The final models included all predictors that were significant for either model. An additional model was explored to examine the relationship between NELP and parent access to developmental services at 24 months.

Analyses were conducted using STATA version 17 (StataCorp. 2021. *Stata Statistical Software: Release 17*. College Station, TX: StataCorp LLC.)

## Results

**FIGURE 1.**
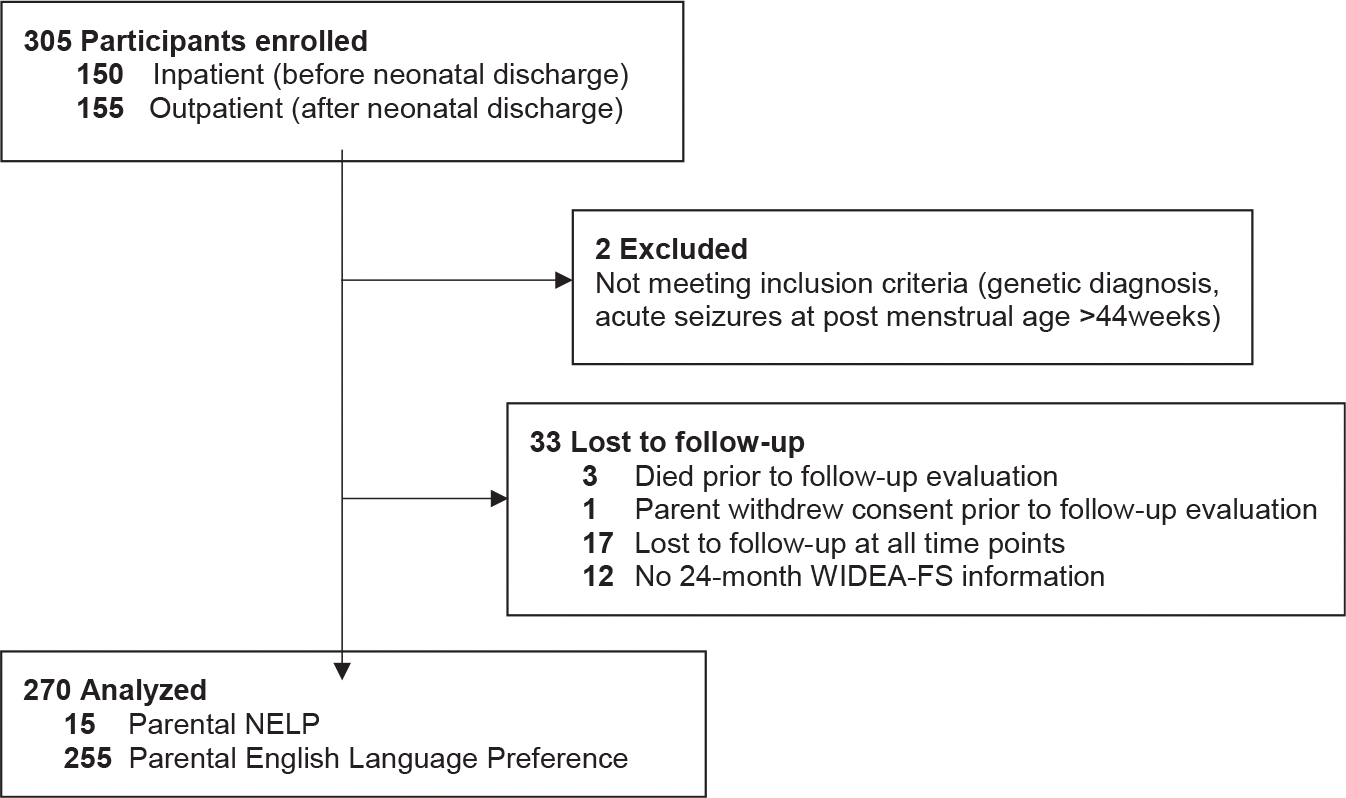
Flow diagram of study participants with acute provoked neonatal seizures.

There were 305 infants enrolled in the *Neonatal Seizure Registry*-II. Two infants were excluded due to not meeting inclusion criteria, and 33 infants were lost to follow-up (Fig 1). Among 270 infants with acute provoked neonatal seizures, 15 (6%) had a parent with NELP (one family had a Chinese language preference; otherwise, all the families had a Spanish language preference). There were no significant differences between groups for key clinical covariates (Table) except for race/ethnicity (*P* < 0.001; a larger proportion of children with parental NELP were Hispanic), formal maternal educational attainment (*P* < 0.001; fewer years among children with parental NELP), and initial EEG pattern (*P* = 0.006; more status epilepticus among infants whose parents prefer English). The primary insurance type, categorized as public or private, was known for 269 children. Most children with parental NELP had public insurance (94%, n = 16), whereas most children with parental English language preference had private insurance (64%, n = 161).

Clinical characteristics of the 33 participants lost to follow-up were not significantly different from those of the enrolled participants (n = 270) other than the likelihood that they were less likely to be hypothermia treated (*P* = 0.04) (Supplementary Table 1). The sociodemographic characteristics of the lost-to-follow-up group differed from that of the enrolled group by the following characteristics: race/ethnicity (*P* = 0.004, participants with NELP were more likely to be Hispanic, less likely to be white), maternal education (*P* < 0.001, generally lower levels), and public insurance (*P* = 0.03, more likely).

In univariate models, we found the following to be significantly associated with WIDEA outcomes: gestational age (β = 3.3, 95% confidence interval [CI]: 2.2 to 4.5), worst EEG background score of severely abnormal (β = −7 referenced to normal, 95% CI −43.4 to −11.0), seizure duration (β = −6.1, 95% CI −8.3 to −3.8), normal neurological examination at discharge (β = 22.6 referenced to abnormal, 95% CI 14.4 to 30.8), complex medical status (β = −21.6 referenced to noncomplex, 95% CI = −30.4 to −12.8), patient was on two or more medications at discharge (β = −19.4 referenced to one or no medication, 95% CI −36.6 to −2.2), child was receiving special services (β = −28.3 compared with not receiving services, 95% CI −36.2 to −20.5), maternal education of college graduate (β = 23.8 referenced to no high school diploma, 95% CI 6.8 to 64.0), and unknown maternal education (β = 35.4 compared with no high school diploma, 95% CI 6.8 to 64.0).

**FIGURE 2.**
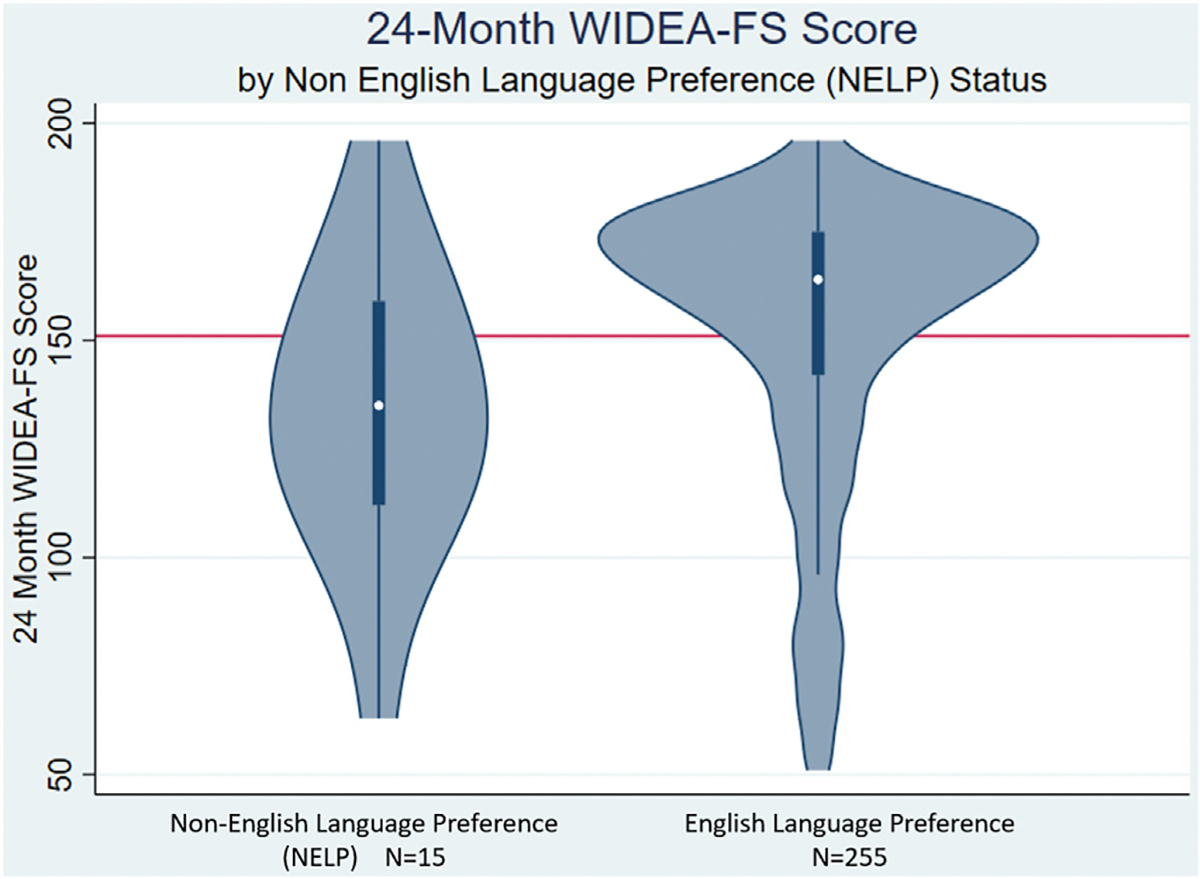
Violin plot of Warner Initial Developmental Evaluation of Adaptive and Functional Skills (WIDEA-FS) at age 24 months in children with parental non-English language preference (NELP) or English language preference. The color version of this figure is available in the online edition.

The covariates included in the final regression models were EEG background, hypothermia treatment, seizure etiology, age at discharge, and neurological examination at discharge. Years of maternal educational attainment was not a significant predictor of functional developmental impairment outcome and was not retained in the final multivariate model, despite its strong association with NELP status as shown in Table. In adjusted analyses, the WIDEA score for infants with parental NELP was on average 13 points (1 S.D.) lower than that for infants without parental NELP; this difference was not statistically significant (95% CI −27 to 1, *P* = 0.08, Fig 2), although there was a trend toward lower WIDEA scores among infants with parental NELP.

In adjusted analyses, children with parental NELP had almost five times the odds of functional developmental impairment on the WIDEA-FS when compared with infants without parental NELP (odds ratio: 4.9, 95% CI: 1.3 to 18.4, *P* = 0.017). The median WIDEA-FS scores in the cohort of infants with parental NELP and parental English language preference were 135 (interquartile range: 112 to 159) and 164 (interquartile range: 142 to 175), respectively. The prevalence of functional developmental impairment on the WIDEA-FS in the total cohort was 34% (91 of 270), with a prevalence in the cohort of infants with parental NELP and parental English language preference of 67% (10 of 15) and 32% (81 of 255), respectively.

Information on access to developmental services was available for 217 children. In a multivariate model including age at discharge, EEG pattern, seizure etiology, and neurological examination at discharge, there was no significant difference in the likelihood of caregiver-reported access to developmental services at 24 months between children with parental NELP and children without parental NELP (odds ratio: 0.9, 95% CI: 0.2 to 3.6, *P* = 0.83).

## Discussion

In this large multicenter cohort of newborns with acute provoked neonatal seizures, children with parental NELP were significantly more likely to have functional developmental impairment based on WIDEA-FS score at 24 months when compared with children without parental NELP. These findings highlight the potential role of language barriers—and associated health inequities—in contributing to developmental outcomes among children with early risk factors for developmental delays.

The results are aligned with previous studies investigating the role of parental NELP, primarily maternal primary language, in the NICU setting.^14–16,22,23^ In a large cohort study of patients from nine NICUs in Massachusetts, maternal primary language other than English was associated with worse NICU outcomes (slower NICU growth, increased incidence of necrotizing enterocolitis, and increased late-onset sepsis), compared with primary English-speaking mothers.^23^ In a single-center study, physicians used interpreters only 39% of the time to provide updates to Spanish-speaking families.^15^ These families were four times more likely to incorrectly identify their child’s diagnosis and self-reported worse understanding of NICU interventions compared with English-speaking parents. In the 48-hour period after discharge, Spanish-speaking parents were less satisfied with their ability to find an NICU doctor or nurse to answer questions.^14^ In another cohort, Spanish-speaking families reported significant differences in their satisfaction with communication compared with English-speaking families.^16^ Spanish-speaking families were less comfortable asking nurses questions, felt less able to participate in decisions regarding the baby, and were less able to participate in the baby’s care.^16^ At one month after discharge in this same cohort, only 32% of Spanish-speaking mothers were aware of early developmental intervention programs despite 55% of study infants being eligible. The current study supports prior studies and highlights the role of parental NELP on a relatively longer-term neurodevelopmental outcome.

To address the role of developmental services in mediating our results, we investigated whether infants with parental NELP had the same perceived access to developmental therapies as their counterparts. There was no significant difference in the likelihood of reporting access to developmental services at age 24 months. This finding suggests that other factors may be contributing to our results, such as the quality and quantity of services, the type of services available (e.g., physical, occupational, or speech therapy), the method of administration of services such as in person or video, and the use of appropriate language resources such as professional interpretation during therapy sessions. Regional differences in the use of therapy services by children with developmental disabilities have been identified.^24,25^ Caregiver-reported access to developmental therapies at age 24 months is a single time point and does not necessarily capture the overall trend of access to therapy over the child’s lifespan; this is a limitation of the study. Additionally, we did not evaluate the type of therapies the infants had access to (or were referred to) and whether infants used the services they had access to.

A child’s insurance may determine the types and the quantity of developmental services available. In our study, most children (93%) with parental NELP had public insurance, whereas 63% of children with parental English language preference had private insurance. It is possible that the families with parental English language preference were able to augment public services with services from privately funded organizations by using either private insurance benefits or other financial or social resources. Despite the mandate that all federally funded public agencies provide therapy services in a primary caregiver’s preferred language, differences in the use of professional interpretation and in the access and quality of language resources may occur. More studies are needed to investigate the factors that contribute to effective and high-quality developmental services in infants with neonatal seizures including language resources.

We recognize that parental NELP is likely a proxy for discriminatory experiences and structural barriers that impact health and socioeconomic opportunities, as it is not a biological construct with plausible pathways impacting brain development. There are a myriad of challenges at the individual, microsystem, and macrosystem levels that may impact quality of care in patients with parental NELP.^26–28^ Children with parental NELP are at risk of poorer communication while in the hospital, and this may have lasting effects outside the hospital. These patients may continue to be at risk with every interaction within their network of care, which may include multiple subspecialists and regional parental and developmental resources.

Our study had limitations, including, most notably, the small number of children with parental NELP. Fifteen children (6%) had parental NELP, mostly with a Spanish language preference. The 2019 US census bureau data 2019 show that 4% of all US households were limited to English-speaking households (defined as households in which there were no members aged 14 years or older who speak only English or speak English “very well”) and approximately 8% of people in the US are considered to have limited English proficiency.^29^ These categories may not fully reflect the families enrolled in this study, where only one of the caregivers was needed to have a non-English primary language and expressed the need for professional interpretation with clinical research staff. The study design mostly enrolled families with Spanish language preference. Therefore the results may not generalize to all children with parental NELP other than Spanish in the United States.

The small number of infants with parental NELP may have affected the ability of the study to detect a significant difference in WIDEA scores. The difference between the WIDEA scores of infants with parental NELP compared with parental English language preference did not meet statistical significance, although there was a trend toward lower WIDEA scores (*P* = 0.08) in the infants with parental NELP. The study may have been underpowered to detect a significant difference.

Another consideration is whether the research study adequately captured parental NELP status. In the literature, there are no unified methods for determining NELP status. In the adult and pediatric literature, parental NELP status has been determined by various methods, including a designation of a non-English language as a preferred language in the electronic medical record, a social work intake documentation noting a non-English preferred language, and an identification of families who have difficulties communicating in English as determined by the health care staff.^15,22,23,30–33^ In pediatrics, there may be multiple caregivers involved during the span of a child’s interactions with the health care team and each of those caregivers may have unique language communication preferences. We did not assess for bilingual status or the language abilities among a child’s primary caregivers. Given the complexity of language skills within a household, further studies are needed to understand the best way to measure effective communication in the medical context among a child’s network of primary caregivers.

Study participation can be significantly influenced by various socioeconomic barriers that may affect enrollment of patients with parental NELP. In a separate, previously published study involving infants enrolled in the *Neonatal Seizure Registry*-II, 55% (n = 167) of the cohort (total n = 303) were enrolled in the childhood follow-up study.^34^ The factors significantly associated with increased enrollment included private insurance, reporting the child’s race as white, and higher maternal education. In our study, the group of children with parental NELP was much more likely to be publicly insured and strongly associated with lower maternal education. Given the overlapping factors between children with parental NELP in our study and children who did not enroll in childhood follow-up in the previous study, greater attention to parental NELP could potentially help in diversifying a study patient population.

There were a few significant differences in the characteristics of infants with parental NELP and those with parental English language preference, including EEG pattern at the onset of recording and specifically electrographic status epilepticus. Twenty-three (9%) infants with parental English language preference had this initial EEG pattern, whereas there were none in the parental NELP group. This difference is most likely related to the low number of infants in the parental NELP group. It is possible that the parental English language preference group had a less severe medical presentation compared with their counterparts; however, there were no other clinical variables that suggested this (i.e., gestational age, Apgar score at five minutes, presence of therapeutic hyperthermia, seizure period, seizure etiology, seizures refractory to initial load dose, two or more antiseizure medications administered, age at discharge, and presence of abnormal neurological examination at discharge).

Two other characteristics that were significantly different among infants with parental NELP and those without were formal maternal educational attainment (*P* < 0.001; fewer years among children with parental NELP) and public insurance (94% of infants with parental NELP publicly insured), which could be potential confounders to the findings related to parental NELP and WIDEA-FS score. In the current study, the authors are unable to further delineate the relationship between neurodevelopmental outcomes and public insurance status or parental NELP, as all the enrolled families with NELP had public insurance.

Additionally, the effect of how the WIDEA-FS is administered is unclear. The WIDEA-FS is available in a Spanish version. In this study, the English version was administered by a clinical research coordinator in the caregiver’s preferred primary language using an interpreter. The translated Spanish version may have nuances that are missed by directly translating the English version in real time; however, a professional interpreter may be able to identify these nuances in real time and adjust the translation accordingly. There are no cross-cultural validity studies of the WIDEA-FS, although it has been used in Spanish-speaking countries such as Colombia.^35^

Further research into the root causes of neurodevelopmental differences will require optimizing research engagement for families with parental NELP and other historically marginalized groups, which may include hiring multilingual clinical research coordinators, allotting more time for professional interpretation at each appointment, and providing professionally translated written materials. At the level of the NICU, there are effective published interventions that improve medical communication with patients and families that target increasing utilization of professional interpretation, which may include language-concordant clinicians.^36^

## Conclusion

Among infants with neonatal seizures, children with parental NELP were more likely to have functional developmental impairment at age 24 months when compared with children without parental NELP. Parental NELP is not a biological construct with plausible pathways affecting brain development and, therefore, is most likely a proxy for structural and discriminatory experiences that impact health and developmental opportunities. A major limitation of the study is the small number of children enrolled with parental NELP (6%, n = 15). Future studies are needed to elucidate the social drivers of health in children with parental NELP to decrease any gaps in neurodevelopmental attainment. Public insurance and lower maternal formal educational attainment are sociodemographic factors that are significantly associated with infants with parental NELP who had functional developmental impairment. The relationship between these variables needs to be better characterized in future studies.

## Supplementary Material

Supplementary Table 1

Supplementary Data

Supplementary data related to this article can be found at https://doi.org/10.1016/j.pediatrneurol.2024.12.010.

## Figures and Tables

**TABLE.** Characteristics of Infants With Acute Provoked Neonatal Seizures and 24-Month Neurodevelopmental Outcomes With and Without Parental NELP

Characteristics	NELP N = 15	English Language Preference N = 255	Total N = 270

Sex: female	5 (33%)	118 (46%)	123 (46%)
Race or ethnicity			
Hispanic (any race)	14 (93%)	23 (9%)	37 (14%)
American Indian/Alaskan Native (non-Hispanic)	0 (0%)	2 (1%)	2 (1%)
Asian (non-Hispanic)	1 (7%)	16 (6%)	17 (6%)
Black or African American (non-Hispanic)	0 (0%)	30 (12%)	30 (11%)
White (non-Hispanic)	0 (0%)	164 (64%)	164 (61%)
More than one race (non-Hispanic)	0 (0%)	7 (3%)	7 (3%)
Other (non-Hispanic)	0 (0%)	5 (2%)	5 (2%)
Unknown/decline to answer	0 (0%)	8 (3%)	8 (3%)
Gestational age at birth (weeks)	39 (37–40)	39 (38–40)	39 (38–40)
Maternal education			
Some education/high school not completed	5 (33%)	9 (4%)	14 (5%)
High school graduate	6 (40%)	32 (13%)	38 (14%)
Some college	2 (13%)	53 (21%)	55 (20%)
College graduate	0 (0%)	91 (36%)	91 (34%)
Graduate study	1 (7%)	62 (24%)	63 (23%)
Unknown/unavailable/declined to answer	1 (7%)	9 (3%)	9 (3%)
Primary insurance type			
Public	14 (93%)	93 (36%)	107 (40%)
Private	1 (7%)	161 (63%)	162 (60%)
Unknown	0 (0%)	1 (0%)	1 (0%)
Apgar score at 5 minutes	8 (5–9)	7 (4–9)	7 (4–9)
Therapeutic hypothermia	4 (27%)	78 (31%)	82 (30%)
EEG pattern at the onset of recording			
Normal	5 (33%)	17 (7%)	22 (8%)
Mild/moderately abnormal	8 (53%)	170 (67%)	178 (66%)
Severely abnormal	2 (13%)	43 (17%)	45 (17%)
Electrographic status epilepticus	0 (0%)	23 (9%)	23 (9%)
Cannot assess	0 (0%)	2 (1%)	2 (1%)
Seizure period (# days with EEG seizures)	1 (0–3)	1 (1–2)	1 (1–2)
Seizure etiology			
Hypoxic-ischemic encephalopathy	4 (27%)	115 (45%)	119 (44%)
Ischemic infarct	8 (53%)	64 (25%)	72 (27%)
Intracranial hemorrhage	1 (7%)	44 (17%)	45 (17%)
Other	2 (13%)	32 (13%)	34 (13%)
Initial antiseizure medication load			
Phenobarbital	12 (80%)	230 (90%)	242 (90%)
Levetiracetam	2 (13%)	13 (5%)	15 (6%)
Phenytoin/fosphenytoin	1 (7%)	2 (1%)	3 (1%)
No loading dose given	0 (0%)	10 (4%)	10 (4%)
Seizures refractory to initial loading dose			
No	5 (33%)	81 (32%)	86 (32%)
Yes	10 (67%)	159 (62%)	169 (63%)
Unknown	0 (0%)	5 (2%)	5 (2%)
No loading dose given	0 (0%)	10 (4%)	10 (4%)
Two or more antiseizure medications administered	7 (47%)	138 (54%)	145 (54%)
Age at discharge (days)	12 (8–41)	15 (9–28)	15 (9–28)
Abnormal neurological examination at discharge	5 (33%)	79 (31%)	84 (31%)

Abbreviations:

EEG = Electroencephalography

NELP = Non-English Language Preference
