# Supplementary material for: Preferred Parental Language and Neurodevelopmental Outcomes Among Infants With Acute Provoked Neonatal Seizures in the United States
Source: Pediatr Neurol. Author manuscript; Available in PMC 2026 Jul 10. (PMC13354519; doi:10.1016/j.pediatrneurol.2024.12.010)
Supplement: Supplementary Table 1 [file NIHMS2184142-supplement-Supplementary_Table_1.docx]

**Supplementary Table 1**: Characteristics of infants lost to follow-up with acute provoked neonatal seizures with and without parental non-English language preference (NELP) as compared to the infants with a 24-month neurodevelopmental outcome who completed the study

|  | Non-English Language Preference  N=15  (Lost to follow-up n=1) | English Language Preference  N=255  (Lost to follow-up n=32) | Total  N=270  (Lost to follow-up n=33) |
| --- | --- | --- | --- |
| Sex - Female | 5 (33%)  0 (0%) | 118 (46%)  10 (31%) | 123 (46%)  10 (30%) |
|  |  |  |  |
| Race or Ethnicity |  |  |  |
| Hispanic (any race) | 14 (93%)  1 (100%) | 23 (9%)  9 (28%) | 37 (14%)  10 (30%) |
| American Indian/Alaskan Native (non-Hispanic) | 0 (0%) | 2 (1%)  2 (6%) | 2 (1%)  2 (6%) |
| Asian (non-Hispanic) | 1 (7%) | 16 (6%)  3 (9%) | 17 (6%)  3 (9%) |
| Black or African American (non-Hispanic) | 0 (0%) | 30 (12%)  5 (16%) | 30 (11%)  5 (15%) |
| White (non-Hispanic) | 0 (0%) | 164 (64%)  11 (34%) | 164 (61%)  11 (33%) |
| More than one race (non-Hispanic) | 0 (0%) | 7 (3%)  1 (3%) | 7 (3%)  1 (3%) |
| Other (non-Hispanic) | 0 (0%) | 5 (2%)  1 (3%) | 5 (2%)  1 (3%) |
| Unknown/Decline to answer | 0 (0%) | 8 (3%)  0 (0%) | 8 (3%)  0 (0%) |
|  |  |  |  |
|  |  |  |  |
| Gestational Age at Birth (weeks) | 39 (37-40)  40 | 39 (38-40)  39 (37-40) | 39 (38-40)  40 (37-40) |
|  |  |  |  |
| Maternal Education |  |  |  |
| Some education/high school not completed | 5 (33%) | 9 (4%)  4 (12%) | 14 (5%)  4 (12%) |
| High school graduate | 6 (40%)  1 (100%) | 32 (13%)  11 (34%) | 38 (14%)  12 (36%) |
| Some college | 2 (13%) | 53 (21%)  6 (19%) | 55 (20%)  6 (18%) |
| College graduate | 0 (0%) | ` | 91 (34%)  6 (18%) |
| Graduate study | 1 (7%) | 62 (24%)  0 (0%) | 63 (23%)  0 (0%) |
| Unknown/unavailable/declined to answer | 1 (7%) | 9 (3%)  5 (16%) | 9 (3%)  0 (0%) |
|  |  |  |  |
| Primary Insurance Type |  |  |  |
| Public | 14 (93%)  1 (100%) | 93 (36%)  20 (62%) | 107 (40%)  21 (64%) |
| Private | 1 (7%) | 161 (63%)  12 (38%) | 162 (60%)  12 (36%) |
| Unknown | 0 (0%) | 1 (0%) | 1 (0%)  0 (0%) |
|  |  |  |  |
| Apgar Score at 5 Minutes | 8 (5-9)  2 | 7 (4-9)  8 (5-9) | 7 (4-9)  8 (5 – 9) |
|  |  |  |  |
| Therapeutic Hypothermia | 4 (27%)  1 (100%) | 78 (31%)  3 (9%) | 82 (30%)  4 (12%) |
|  |  |  |  |
| EEG Pattern at the Onset of Recording |  |  |  |
| Normal | 5 (33%) | 17 (7%)  3 (9%) | 22 (8%)  3 (9%) |
| Mild/Moderately abnormal | 8 (53%)  1 (100%) | 170 (67%)  20 (59%) | 178 (66%)  21 (64%) |
| Severely Abnormal | 2 (13%) | 43 (17%)  8 (24%) | 45 (17%)  8 (24%) |
| Electrographic Status Epilepticus | 0 (0%) | 23 (9%)  1 (3%) | 23 (9%)  1 (3%) |
| Cannot Assess | 0 (0%) | 2 (1%) | 2 (1%)  0 (0%) |
|  |  |  |  |
| Seizure Period (# days with EEG seizures) | 1 (0-3)  0 | 1 (1-2)  1 (1-2) | 1 (1-2)  1 (1-2) |
|  |  |  |  |
| Seizure Etiology |  |  |  |
| Hypoxic-Ischemic Encephalopathy | 4 (27%)  1 (100%) | 115 (45%)  10 (31%) | 119 (44%)  11 (33%) |
| Ischemic Infarct | 8 (53%) | 64 (25%)  7 (22%) | 72 (27%)  7 (21%) |
| Intracranial hemorrhage | 1 (7%) | 44 (17%)  10 (31%) | 45 (17%)  10 (30%) |
| Other | 2 (13%) | 32 (13%)  5 (16%) | 34 (13%)  5 (15%) |
|  |  |  |  |
| Initial Anti-Seizure Medication Load |  |  |  |
| Phenobarbital | 12 (80%)  1 (100%) | 230 (90%)  30 (88%) | 242 (90%)  31 (94%) |
| Levetiracetam | 2 (13%) | 13 (5%)  2 (6%) | 15 (6%)  2 (6%) |
| Phenytoin/Fosphenytoin | 1 (7%) | 2 (1%)  0 (0%) | 3 (1%)  0 (0%) |
| No Loading Dose Given | 0 (0%) | 10 (4%)  0 (0%) | 10 (4%)  0 (0%) |
|  |  |  |  |
| Seizures Refractory to Initial  Loading Dose |  |  |  |
| No | 5 (33%)  1 (100%) | 81 (32%)  15 (47%) | 86 (32%)  16 (48%) |
| Yes | 10 (67%) | 159 (62%)  17 (53%) | 169 (63%)  17 (51%) |
| Unknown | 0 (0%) | 5 (2%) | 5 (2%)  0 (0%) |
| No Loading Dose Given | 0 (0%) | 10 (4%) | 10 (4%)  0 (0%) |
|  |  |  |  |
| Two or More Anti-Seizure Medications Administered | 7 (47%)  0 (0%) | 138 (54%)  15 (47%) | 145 (54%)  15 (45%) |
|  |  |  |  |
| Age at Discharge (days) | 12 (8-41)  9 | 15 (9-28)  20 (9-46) | 15 (9-28)  20 (9-42) |
|  |  |  |  |
| Abnormal Neurological Exam at Discharge | 5 (33%)  0 (0%) | 79 (31%)  12 (38%) | 84 (31%)  12 (36%) |
|  |  |  |  |

Data are presented as median (interquartile range) for continuous measures, and n (%) for categorical measures.
